# Identification and verification of ferroptosis-related genes in the synovial tissue of osteoarthritis using bioinformatics analysis

**DOI:** 10.3389/fmolb.2022.992044

**Published:** 2022-08-29

**Authors:** Lin Xia, Ningji Gong

**Affiliations:** ^1^ Department of Plastic Surgery, Qilu Hospital, Cheeloo College of Medicine, Shandong University, Jinan, China; ^2^ Department of Emergency, The Second Hospital, Cheeloo College of Medicine, Shandong University, Jinan, China

**Keywords:** osteoarthritis, synovitis, ferroptosis, bioinformatics analysis, differentially expressed genes, ferroptosis DEGs

## Abstract

**Background:** Osteoarthritis (OA) is a major factor causing pain and disability. Studies performed to date have suggested that synovitis is possibly a critical OA-related pathological change. Ferroptosis represents a novel type of lipid peroxidation-induced iron-dependent cell death. However, its effect on OA remains largely unclear.

**Objective:** This work focused on identifying and validating the possible ferroptosis-related genes (FRGs) involved in synovitis of OA through bioinformatics analysis.

**Materials and Methods:** The microarray dataset GSE55235 was downloaded in the database Gene Expression Omnibus (GEO). By the Venn diagram and GEO2R, differentially expressed genes (DEGs) and ferroptosis DEGs (FDEGs) were detected. DEGs were screened by GO and KEGG enrichment analysis, as well as protein-protein interaction (PPI) analysis. Besides, the software Cytoscape and database STRING were utilized to construct hub gene networks. Moreover, this study used the database NetworkAnalyst to predict the target miRNAs of the hub genes. Finally, the hub genes were confirmed by analysis of the receiver operating characteristic (ROC) curve on the GSE12021 and GSE1919 databases. Considering the relationship between ferroptosis and immunity, this study applied CIBERSORTx to analyze the immune infiltration in OA in addition.

**Results:** This work discovered seven genes, including ATF3, IL6, CDKN1A, IL1B, EGR1, JUN, and CD44, as the hub FDEGs. The ROC analysis demonstrated that almost all hub genes had good diagnostic properties in GSE12021 and GSE 1919.

**Conclusion:** This study discovered seven FDEGs to be the possible diagnostic biomarkers and therapeutic targets of synovitis during OA, which sheds more light on the pathogenesis of OA at the transcriptome level.

## 1 Introduction

Osteoarthritis (OA) refers to the chronic joint degenerative disorder that is featured by synovial inflammation, cartilage degeneration, and reduced joint function (Kraus et al., 2015). As a result, patients experience symptoms such as swelling, pain, or stiffness, thereby negatively affecting their quality of life (Roseti et al., 2019). OA is associated with an increased morbidity rate in the last few decades because of the aging population and obesity (Nelson, 2018). According to a recent study, there will be about 400 million OA cases in China by 2030 (Zhang et al., 2020). OA does not exhibit any early symptoms, and none of the existing methods can sensitively and efficiently detect minor changes (Camacho-Encina et al., 2019). Therefore, the identification of biomarkers with high sensitivity and efficiency can contribute to the early diagnosis of OA and facilitate the study of the pathogenesis of OA in individualized treatments.

Ferroptosis, an iron-dependent oxidative type of cell death, is different from necrosis, autophagy, apoptosis, or other types of cell death (Dixon et al., 2012). Besides, it is characterized by the accumulation of lipid peroxidation in an iron-dependent manner, which is reflected by aberrant lipid oxide metabolism under the catalysis of excessive ferrous ions, generation of reactive oxygen species (ROS), and cell death mediated by the excessive oxidation of polyunsaturated fatty acid (PUFA). Recently, ferroptosis has become the hotspot for research on various types of disease, such as heart injury, brain injury, cancer, asthma, or acute kidney failure (Friedmann Angeli et al., 2014; Stockwell et al., 2017; Zhao et al., 2020). Ferroptosis and iron have been recently suggested to have important effects on the occurrence of OA (Yao et al., 2021; Miao et al., 2022), although their exact mechanism remains largely unclear. Since studies have been increasingly conducted to explore ferroptosis and its relation with some biological processes, targeting ferroptosis, either used alone or as adjuvant therapy, can be a feasible approach to treat OA (Qu et al., 2022). Therefore, it is necessary to further analyze ferroptosis-related genes (FRGs) that are related to synovial inflammation, which may provide potential therapeutic targets.

This study obtained data from OA cases and healthy subjects from the database Gene Expression Omnibus (GEO) for bioinformatics analysis with the purpose of screening the differentially expressed genes (DEGs). Then, the identified DEGs were intersected with the ferroptosis dataset with the purpose of obtaining the key FDEGs. By constructing the protein-protein interaction (PPI) network, the current work detected certain hub FDEGs as the key regulating factors for the occurrence of OA, which were the potential targets. These key FDEGs were then exposed to Gene Ontology (GO) and Kyoto Encyclopedia of Genes and Genomes (KEGG) analysis. Thereafter, levels of these genes were analyzed in the GSE12021 and GSE1919 datasets. As demonstrated by the ROC curve, seven key genes had good diagnostic properties. Finally, given the potential relationship between ferroptosis and immunity, we used CIBERSORTx to analyze the immune infiltration in OA. This work may offer more insight into exploring the mechanism of ferroptosis in OA together with the diagnosis and treatment of OA.

## 2 Materials and methods

### 2.1 Data collection

The GSE55235, GSE12021 and GSE1919 datasets were acquired from the GEO database (https://www.ncbi.nlm.nih.gov/geo/). In addition, we downloaded the FRGs from the FerrDb online database (http://www.zhounan.org/ferrdb/).

### 2.2 Identification of differentially expressed genes

The DEGs were screened and identified based on the program GEO2R (https://www.ncbi.nlm.nih.gov/geo/geo2r) in GSE55235. Besides, the DEGs were defined as |log FC|>1 (*p* < 0.05). The FDEGs were defined as DEGs from GSE55235 that overlapped with FRGs and were selected based on the Venn diagram.

### 2.3 GO functional annotation and kyoto encyclopedia of genes and genomes analysis

The program DAVID (https://david.ncifcrf.gov/, version 6.8) was employed with the purpose of performing GO functional annotation and KEGG analysis, with the threshold set at *p* < 0.05. Furthermore, the GO terms were divided into three categories, including biological processes (BPs), molecular functions (MFs) as well as cellular components (CCs).

### 2.4 Identification of gene clusters and construction of protein-protein interaction network

The STRING database (https://string-db.org/) was applied to construct the PPI network in order to obtain the diagram. In addition, the results were obtained in STRING online database and were then imported into the software Cytoscape v 3.9.1 to choose the critical nodes for the visualization of molecular interaction networks. Based on our constructed PPI network, the key genes were identified by the CytoHubba plugin.

### 2.5 Key gene-related miRNAs

With the aim of constructing the miRNA-gene interactions for the identified key genes, the online software NetworkAnalyst (version 3.0, https://www.networkanalyst.ca/) was applied.

### 2.6 Validation of the hub genes with receiver operating characteristic

The diagnostic accuracy of the FDEGs was analyzed by ROC curves in the dataset GSE12021 and GSE 1919.

### 2.7 Immune cell infiltration analysis using CIBERSORTx

To evaluate the function of immune microenvironment in OA formation, CIBERSORTx (https://cibersortx.stanford.edu/) was adopted for analyzing the immune infiltration differences between OA and normal groups. Then, the results were visualized by a bar-plot and box-plot.

### 2.8 Statistical analysis

The statistical software R version 4.2.0 was applied to perform the statistical analysis, with p-values of <0.05 suggesting statistical significance.

## 3 Results

### 3.1 Identification of differentially expressed genes and ferroptosis differentially expressed genes

**FIGURE 1:**
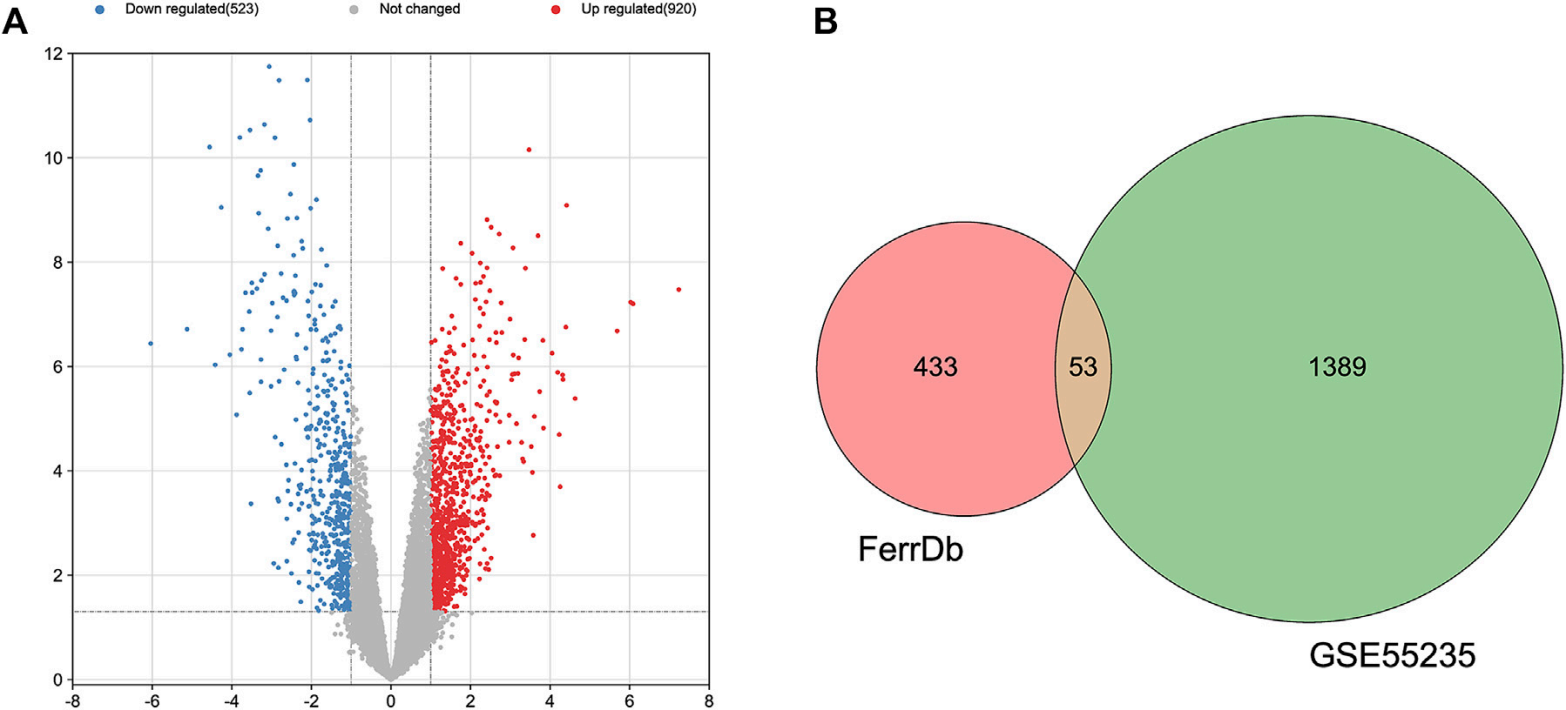
Identification of DEGs. **(A)** A volcano plot showing DEGs in the dataset GSE55235. **(B)** After the intersection, 53 FDEGs were identified based on the Venn diagram.

**FIGURE 2:**
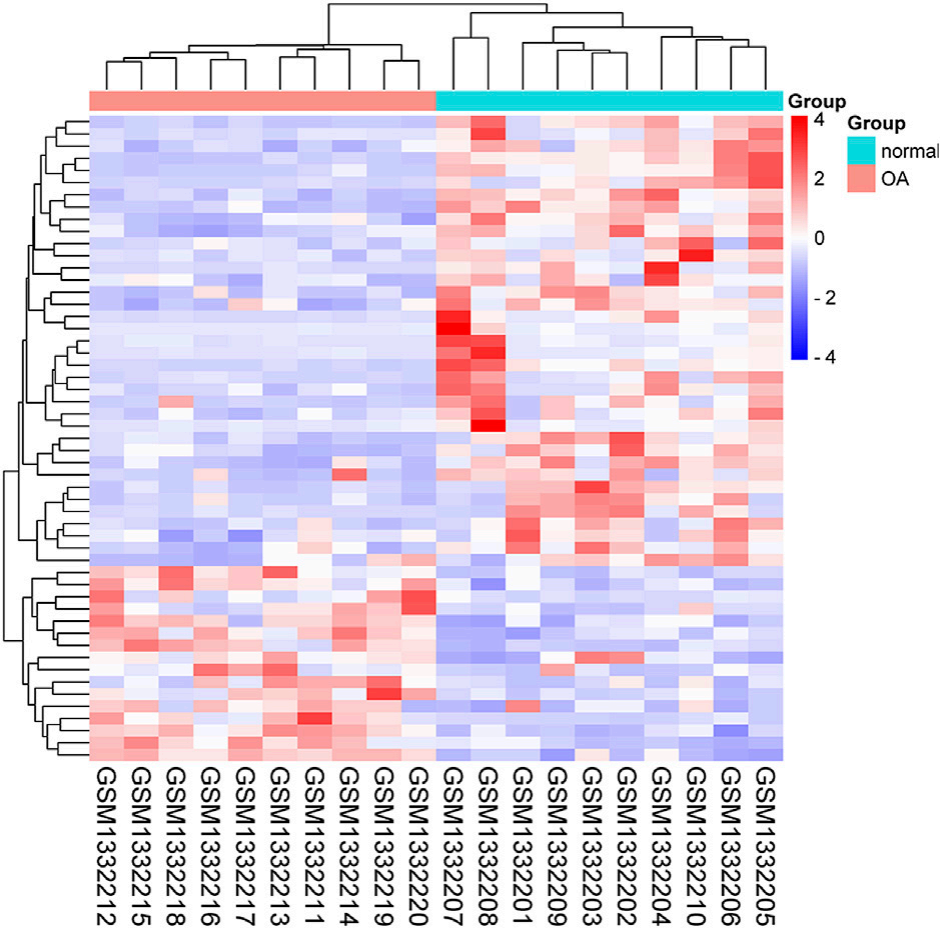
Heatmap of the 53 FDEGs.

The GEO2R is a web-based tool that is specialized in analyzing DEGs in the OA group in relative to the healthy group. Besides, the two groups were compared using GEO2R in the GEO datasets GSE55235 and the results were downloaded for further analysis. Genes of |log FC| > 1 and *p* < 0.05 were identified as DEGs. Totally 1,443 DEGs, containing 920 upregulated and 523 downregulated DEGs were detected. A volcano plot was drawn to validate the results (Figure 1A). These DEGs were intersected with 486 FRGs downloaded from the FerrDb database (http://www.zhounan.org/ferrdb/). Finally, 53 FDEGs were selected on the basis of the Venn diagram **(** Figure 1B). A heatmap of the 53 FDEGs is presented in Figure 2.

### 3.2 GO function and kyoto encyclopedia of genes and genomes pathway analyses of ferroptosis differentially expressed genes

**FIGURE 3:**
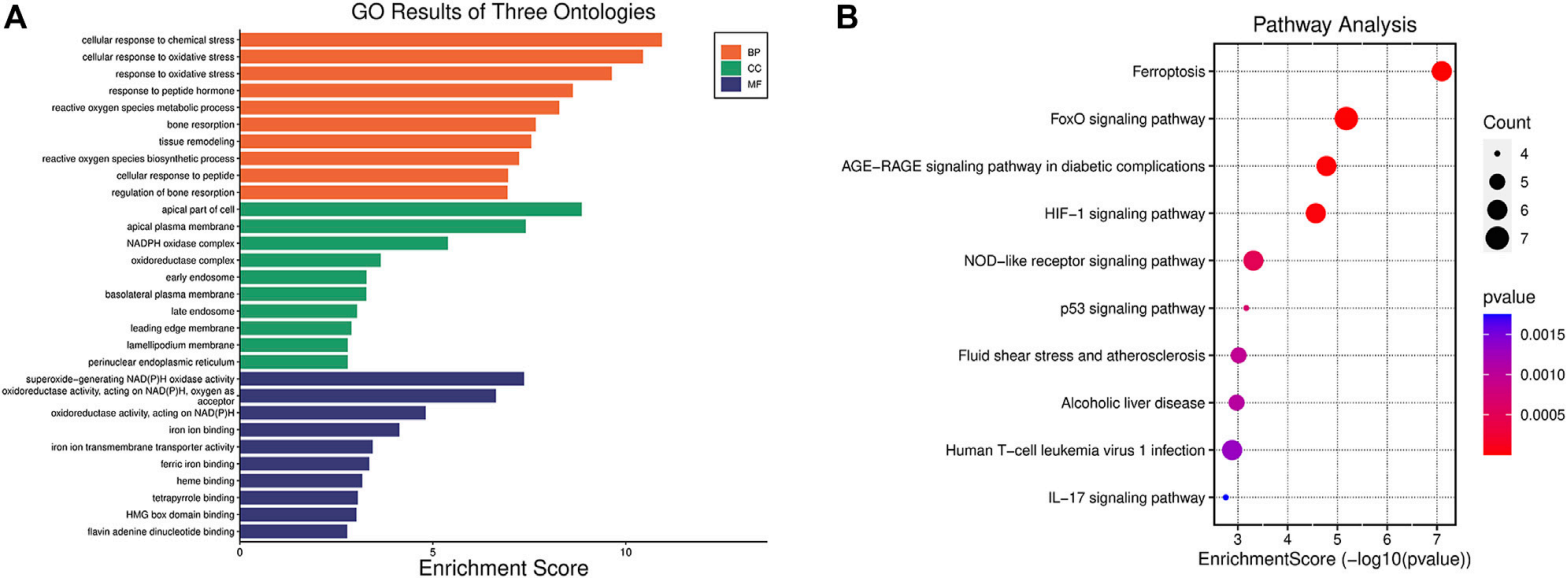
Functions and pathways explained by the 53 FDEGs. **(A)** GO enrichment analysis of the 53 FDEGs with bar-plot. **(B)** Results of the KEGG pathway analyses of the 53 FDEGs with dot-plot.

With the purpose of investigating the biological activities and pathways associated with FDEGs among the OA cases, GO and KEGG enrichment analyses (Yu et al., 2012; Luo and Brouwer, 2013) were performed on the 53 genes. Besides, the significantly enriched GO terms were cellular response to chemical stress, cellular response to oxidative stress (OS), response to OS (BP); apical part of the cell, apical plasma membrane, NADPH oxidase complex (CC); superoxide-generating NADA(P)H oxidase activity, oxidoreductase activity, acting on NAD(P)H, oxygen as acceptor, iron ion binding (MF) (Figure 3A). As revealed by the KEGG enrichment analysis, the 53 FDEGs were mostly associated with ferroptosis, FoxO pathway, AGE-RAGE pathway in diabetic complications, and HIF-1 signaling pathway (Figure 3B).

### 3.3 Protein-protein interaction network analysis and screening of key genes for differentially expressed genes associated with ferroptosis

**FIGURE 4:**
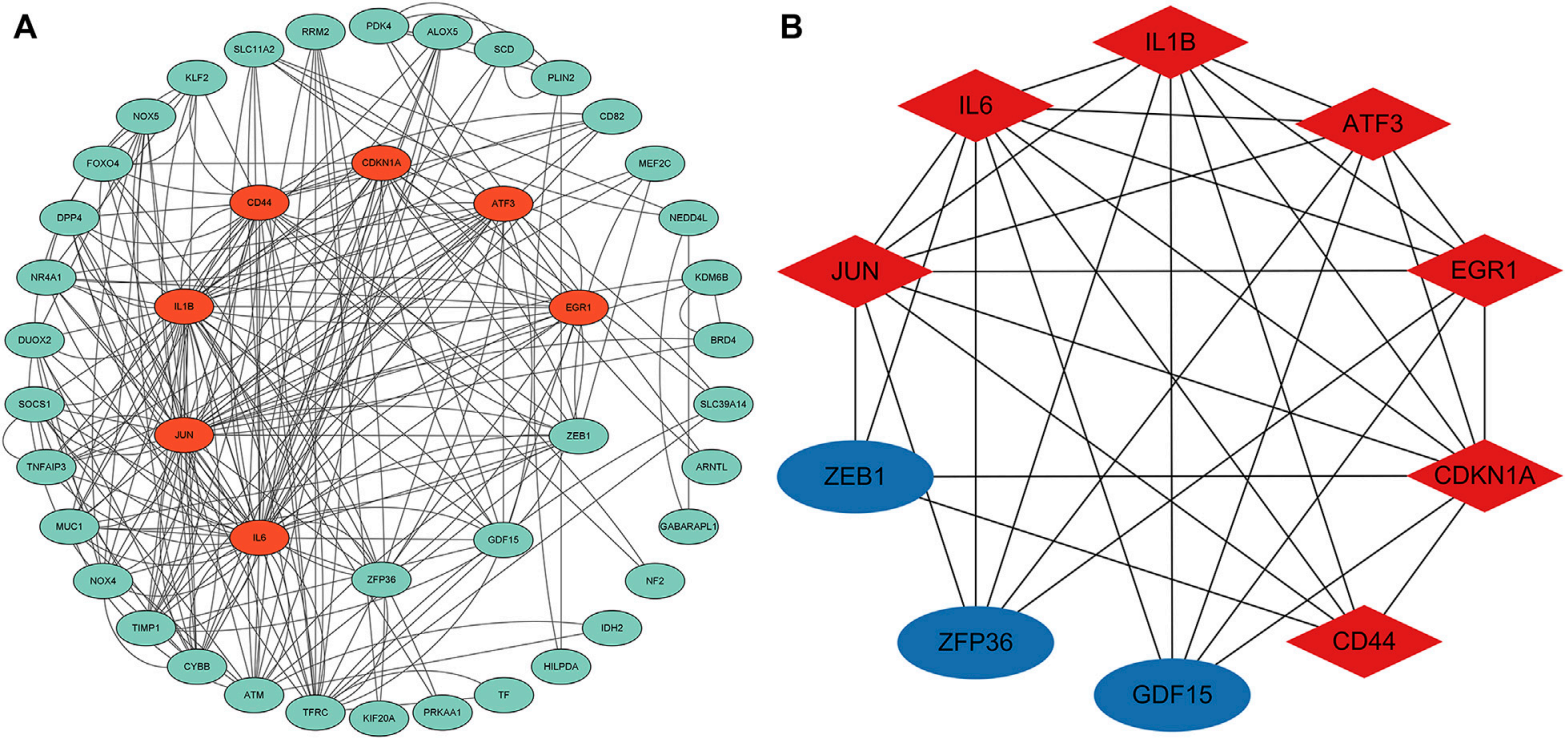
PPI analysis of the 53 FDEGs. **(A)** Cytoscape network visualization of the 53 FDEGs. **(B)** Network visualization of the 10 key genes by adopting the MCC algorithm of the CytoHubba plugin. Red and blue nodes represent the first-and second-level key genes, respectively.

To analyze the PPI network of the 53 FDEGs, the database STRING (version 11.5, http://cn.string-db.org/) was applied. Cytoscape network visualization was obtained on the basis of the STRING database (Figure 4A). Using the MCC algorithm, 10 hub genes were obtained through the CytoHubba plugin of the software Cytoscape. Subsequently, according to the rank score, seven first-level hub genes, including ATF3, IL6, CDKN1A, IL1B, EGR1, JUN, and CD44 were identified with red nodes (Figure 4B; Table 1; Supplementary Table S2).

**TABLE 1:** The seven first-level hub genes.

Gene symbol	LogFC	Change	*p*-value	FerrDb
ATF3	−3.1775742	down	1.71E-08	driver
IL6	−2.8234462	down	0.000381	driver
CDKN1A	−2.40141	down	1.82E-08	suppressor
IL1B	−2.3712649	down	0.000000728	driver
EGR1	−2.1305895	down	0.0000159	driver
JUN	−2.0392066	down	0.0000051	suppressor
CD44	−1.8051573	down	0.000873	suppressor

### 3.4 Kyoto encyclopedia of genes and genomes analysis enriched by key ferroptosis differentially expressed genes

**FIGURE 5:**
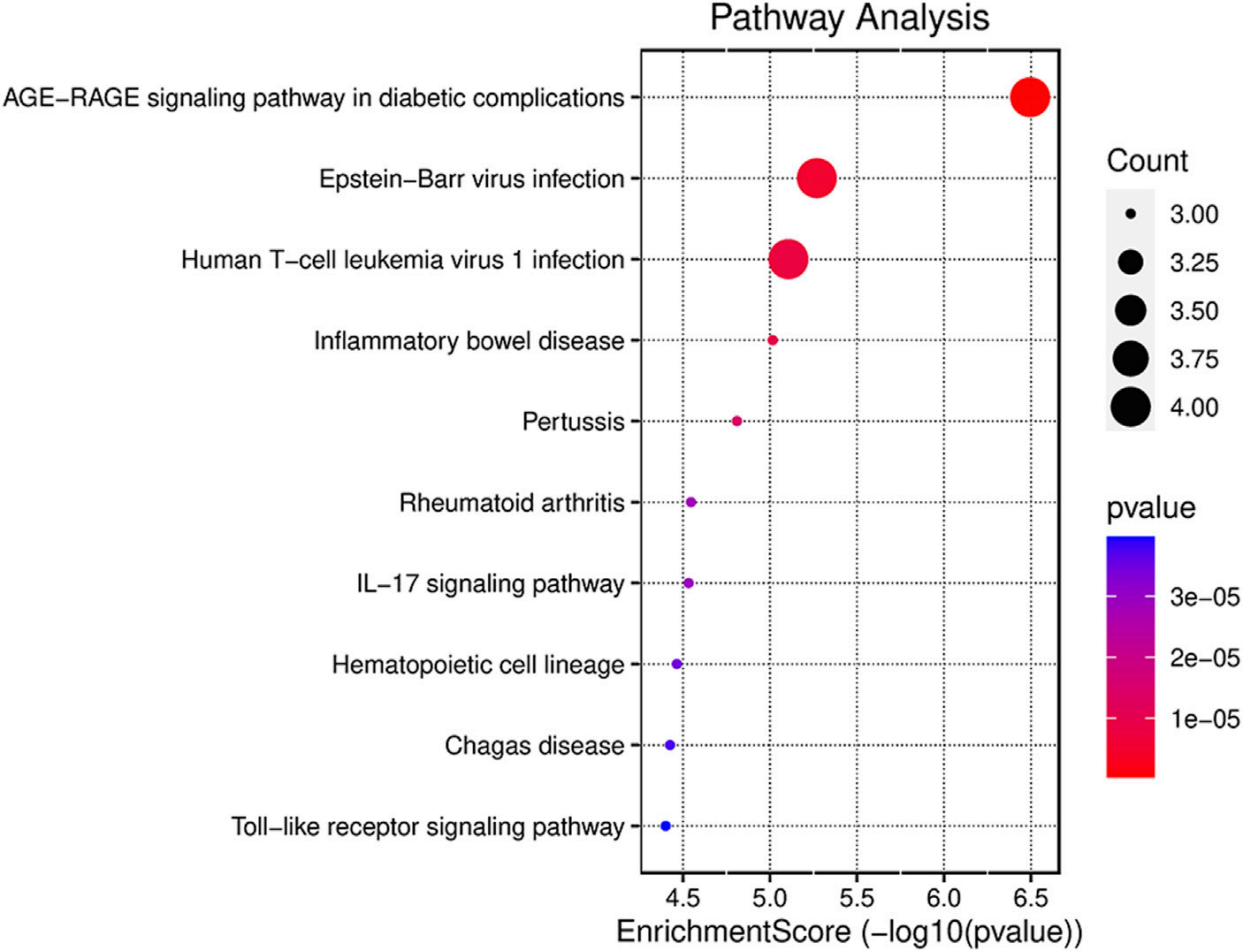
Results of the KEGG pathway analyses of the seven hub FDEGs with dot-plot.

To illustrate the possible pathways of the seven hub FDEGs, KEGG analyses were performed. On the basis of the KEGG analysis, these hub FDEGs were mostly related to the AGE-RAGE signaling pathway and Epstein-Barr virus infection (Figure 5).

### 3.5 Estimation of target miRNAs and establishment of the miRNAs-Targets network

**FIGURE 6:**
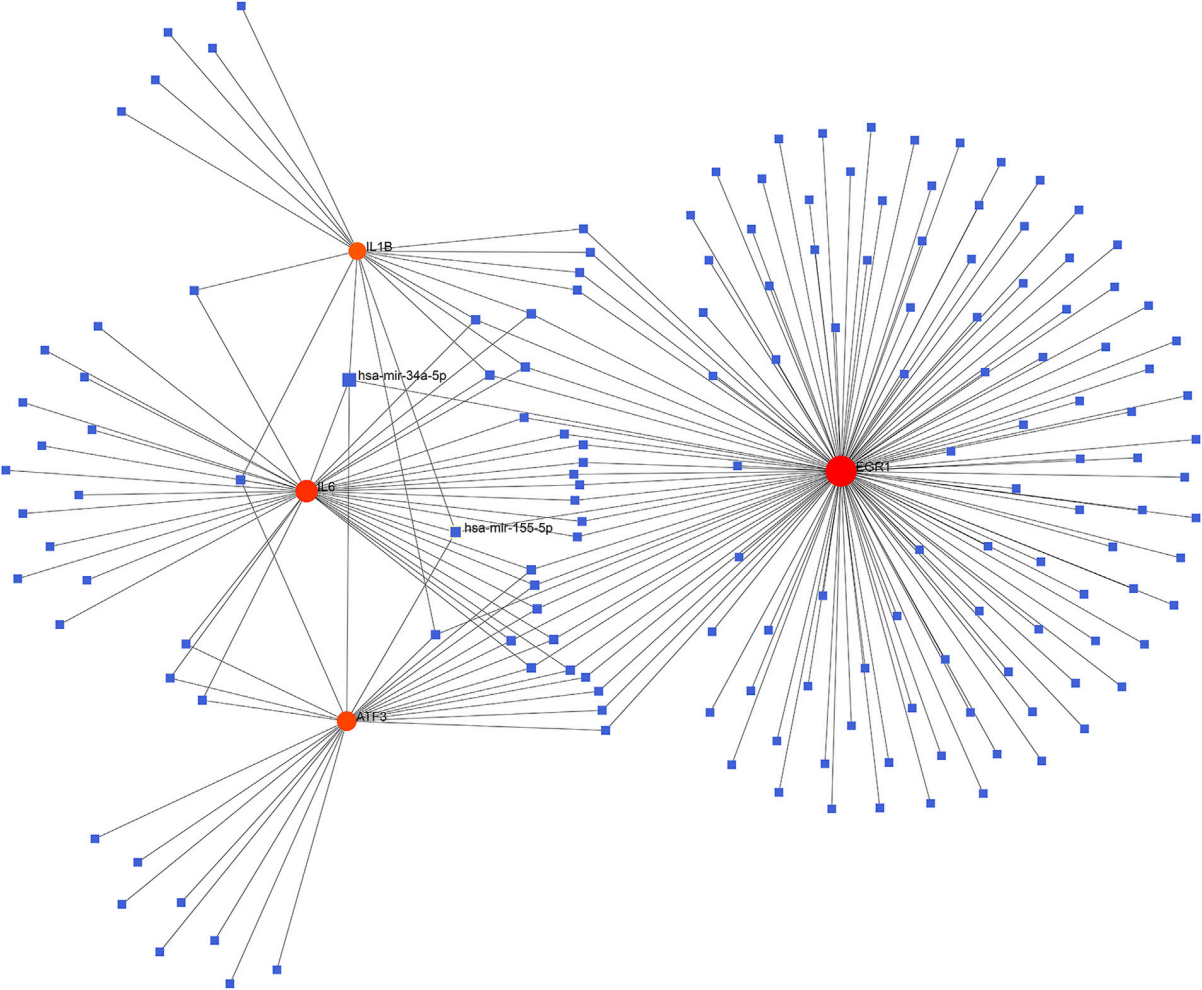
Establishment of the miRNA-gene interaction networks based on the four ferroptosis-driver key FDEMs. Red circles and blue squares represent key genes and miRNAs related to key FDEGs.

The database NetworkAnalyst was adopted for predicting the target miRNA of the key genes. The possible miRNA-hub gene network was constructed to accurately investigate the molecular mechanism underlying the four ferroptosis-driver FDEGs. The miR-34a-5p and miR-155-5p interacted with all the four FDEMs, namely, ATF3, IL6, IL1B, and EGR1 (Figure 6).

### 3.6 Validation of key genes from the GSE12021 and GSE1919 datasets

**FIGURE 7:**
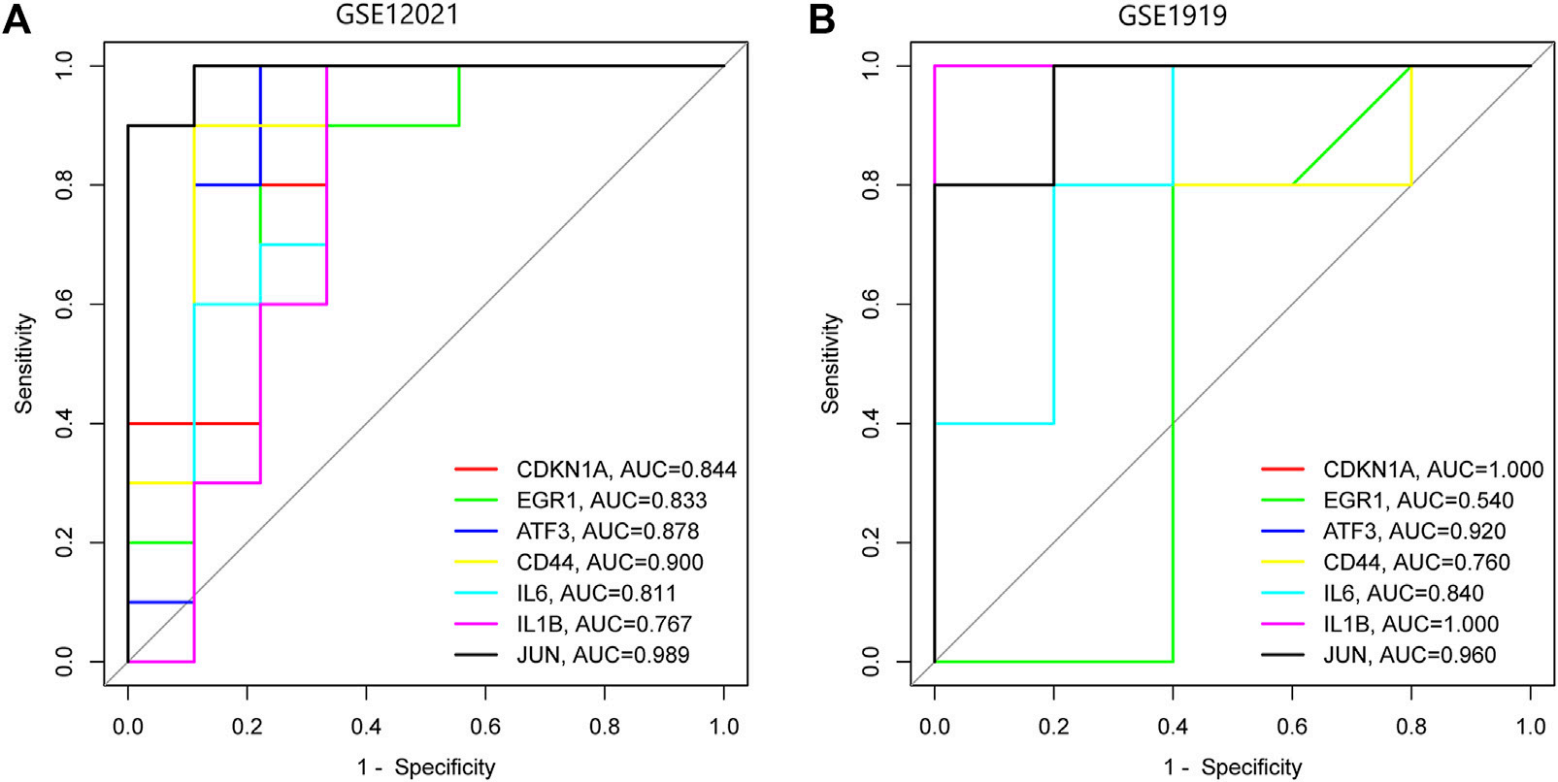
ROC analysis of seven key FDEGs. **(A)** on the GSE12021 dataset. **(B)** on the GSE1919 dataset.

To verify whether the above seven key FDEGs had predictive significance, an analysis of the ROC curve was performed on the GSE12021 and GSE1919 datasets. It was observed that the AUCs of almost all hub genes were greater than 0.75 (Figure 7; Supplementary Table S1).

### 3.7 Immune cell infiltration analysis by CIBERSORTx

**FIGURE 8:**
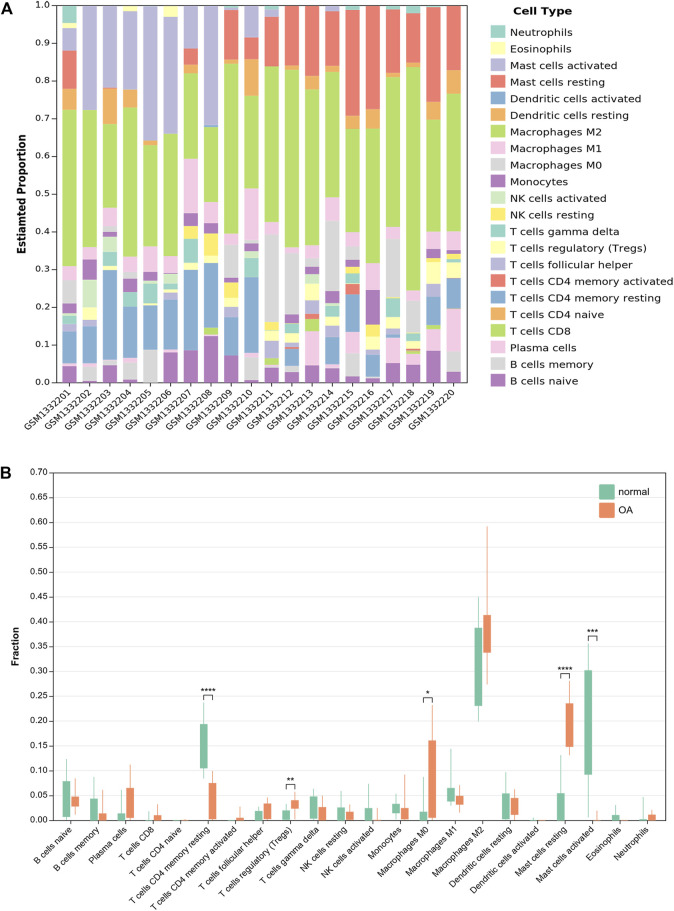
Results of immune infiltration by CIBERSORTx. **(A)** Bar-plot showed the composition of 22 kinds of immune cells in GSE55235. **(B)** Box-plot presented the difference of immune infiltration of 22 kinds of immune cells.

CIBERSORTx was used to identify the types of immune cells involved in the formation of OA. We obtained the proprotions of 22 kinds of immune cell types between OA and normal synovium. Compared with normal synoviums, the proportions of T cells regulatory (Tregs), macrophages M0 and mast cells resting were relatively high, while T cells CD4 memory activated and mast cells activated were low (Figure 8; Supplementary Table S3).

## 4 Discussion

Previously, researchers focused mainly on articular cartilage while ignoring the function of soft tissues surrounding knee joints in the occurrence of OA (Trachana et al., 2019). Recently, increasing studies have suggested that synovitis may also occur during the early to late stage of OA (Wang et al., 2018). Therefore, studying the mechanism of synovitis is vital. However, little is known about the mechanism of synovitis in OA (Neumann et al., 2002). Moreover, while ferroptosis is extensively investigated in various tumor types (Wang et al., 2016; Zhang et al., 2018), its function in synovitis of OA is much less clear.

This work identified the molecular signatures of ferroptosis that were associated with synovitis of OA by analyzing the DEGs in OA cases compared to healthy subjects. Finally, 53 FDEGs were acquired by intersecting GSE55235 with FerrDb. Furthermore, GO and KEGG analyses were conducted in order to investigate the GO terms and pathways enriched by these 53 FDEGs. The oxidative stress and NADA(P)H oxidase were significantly enriched by these FDEGs, suggesting that these FDEGs were related to oxidation, which agreed with previous results (Shen et al., 2021). Through KEGG pathway enrichment, we observed that the genes were mostly enriched in the FoxO and HIF-1 signaling pathway. FoxO regulated the cell-aging mechanisms, such as defense against OS and autophagy (van der Horst and Burgering, 2007). Besides, the activation and level of FoxO are associated with OA and aging (Akasaki et al., 2014). HIF-1α is a major transcription factor regulating the developmental and cellular responses to hypoxia. The loss of HIF-1α has been shown to aggravate the level of MMP13 and degrade cartilage tissue (Bouaziz et al., 2016). Our results helped describe the aging and OS-related pathways, which might expand our understanding of the mechanisms of OA.

Furthermore, we examined the PPI results using the program cytoHubba in Cytoscape, which revealed seven key FDEGs, namely, ATF3, IL6, CDKN1A, IL1B, EGR1, JUN, and CD44. They have very high diagnostic efficiency in GSE12021 and GSE 1919. Furthermore, this work conducted KEGG analysis on the seven hub FDEGs. The results indicated that these hub FDEGs were mostly associated with AGE-RAGE pathway and the Epstein-Barr virus infection. Epstein-Barr virus was reported to show association with rheumatoid arthritis (Balandraud and Roudier, 2018). However, there are still few reports about Epstein-Barr virus infection and OA. According to our findings, this pathway is possibly related to ferroptosis-dependent synovial hyperplasia. However, further research is needed.

MiRNAs have been reported to be related to OA, and several miRNAs have been implicated in disease pathogenesis (Zhang et al., 2021). This work also established the miRNA-target gene network, based on which, miR-34a-5p and miR-155-5p interacted with all the four ferroptosis-driver hub FDEMs (ATF3, IL6, IL1B and EGR1) were selected. Mir-34a-5p expression is significantly increased in the synovium of patients with advanced OA, but the underlying mechanisms need to be further elucidated (Endisha et al., 2021). Recent research has found that mir-34a-5p targeting SIRT1 induces cell ferroptosis (Hao et al., 2022). But its role in OA is still unclear. Mir-155-5p was found to be related to OA (Soyocak et al., 2017), and it promoted polarization of macrophages M1 and reduced apoptosis of macrophages (Li et al., 2021). According to the results of our study, these miRNAs might participate in the ferroptosis of the synovium. However, their roles in regard to ferroptosis in OA synovial hyperplasia needs to be further clarified.

Ferroptosis has been reported to be associated with immune diseases such as rheumatoid arthritis (Zhao et al., 2022). Nevertheless, the exact mechanism between ferroptosis and immunity remains unclear. So, we used CIBERSORTx in order to investigate the infiltration of immune cells in OA synovium. The results showed that some immune cells in OA synovium were significantly different from normal synoviums. Compared with normal synoviums, the proportions of T cells regulatory (Tregs), macrophages M0 and mast cells resting were relatively high, while T cells CD4 memory activated and mast cells activated were low. However, the exact mechanism of their interaction remains to be further studied.

However, this study still has some limitations. At first, this work used a small sample size, which might induce bias. Second, more *in-vivo* and *in-vitro* studies were required to verify the diagnostic and prognostic significance of these factors. Finally, a comprehensive understanding of the hub-gene target of OA is necessary for the discovery and the progress of appropriate therapeutic strategies.

## 5 Conclusion

Our study identified seven hub FDEGs as possible diagnostic biomarkers and therapeutic targets for synovitis of OA. These seven hub genes (including ATF3, IL6, CDKN1A, IL1B, EGR1, JUN and CD44) were significantly downregulated. Among them, four were ferroptosis-driver genes, which were possibly associated with synovial hyperplasia. This study also predicted certain possible target miRNAs (miR-34a-5p and miR-155-5p), which might be related to the pathophysiological process of OA *via* ferroptosis. In addition, our analysis confirmed differences in immune infiltration between OA and normal specimens. Moreover, this study may offer more insight into the identification of markers for the diagnosis and treatment of OA.

## Data Availability

The original contributions presented in the study are included in the article/Supplementary Material, further inquiries can be directed to the corresponding author.
